# Biotransformation of Glycosylated Saponins in Balloon Flower Root Extract into 3-*O*-β-D-Glucopyranosyl Platycosides by Deglycosylation of Pectinase from *Aspergillus aculeatus*

**DOI:** 10.4014/jmb.2001.01041

**Published:** 2020-03-13

**Authors:** Jung-Hun Ju, Su-Hwan Kang, Tae-Hun Kim, Kyung-Chul Shin, Deok-Kun Oh

**Affiliations:** Department of Bioscience and Biotechnology, Konkuk University, Seoul 05029, Republic of Korea

**Keywords:** Balloon flower root extract, platycosides, pectinase, 3-*O*-β-D-Glucopyranosyl platycosides, biotransforamtion

## Abstract

*Platycodon grandiflorum* root (Platycodi radix) saponins, platycosides, have been used as health supplements and food items for the treatment of respiratory disorders and pulmonary diseases. Deglycosylated saponins have been known to exert stronger biological effects than their glycosylated forms. In the present study, glycosylated platycosides in Platycodi radix extract were biotransformed into deglycosylated 3-*O*-β-D-glucopyranosyl platycosides, including 3-*O*-β-Dglucopyranosyl platycodigenin, 3-*O*-β-D-glucopyranosyl polygalacic acid, and 3-*O*-β-Dglucopyranosyl platyconic acid, by pectinase from *Aspergillus aculeatus*. This is the first report on the quantitative enzymatic production of 3-*O*-β-D-glucopyranosyl platycosides. The chemical structures of 3-*O*-β-D-glucopyranosyl platycosides were identified with LC/MS. Moreover, the biotransformation pathways of the three types of platycosides in Platycodi radix into 3-*O*-β-Dglucopyranosyl platycosides were established.

## Introduction

**Fig. 1:**
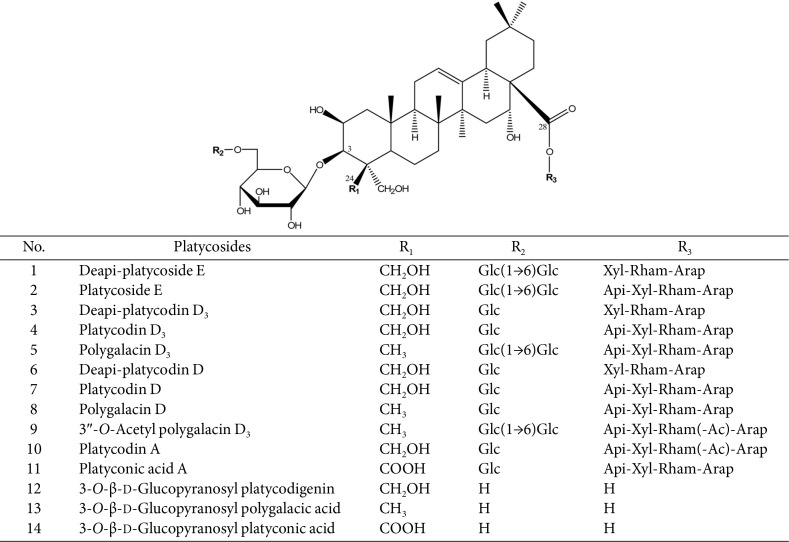
Chemical structures of triterpenoid platycosides from Platycodi radix extract and platycosides obtained from the biotransformation with pectinase from *A. aculeatus*. Glycosylated platycosides from Platycodi radix extract are numbers **1-11**. The products, 3-*O*-β-D-glucopyranosyl platycosides, are numbered **12-14**. Glycosylated platycosides from Platycodi radix extract contain glycosides at C-3 and C-28. The glycosides at C-3 are Glc, Glc-Glc, and Glc- Glc-Glc, while those at C-28 are Ara-Rham (or Rham(Ac))-Xyl-Api. Glc, β-D-glucopyranosyl-; Ara, α-L-arabinopyranosyl-; Rham, α-L-rhamnopyranosyl-; Xyl, β-D-xylopyranosyl-; Api, β-D-apiofuranosyl-; and Ac, acetyl.

*Platycodon grandiflorum* A.DC. (Campanulaceae), commonly known as “balloon flower” or “bell flower,” is a perennial flowering plant widespread in Northeast Asia. *P. grandiflorum* root (Platycodi radix) is used in the preparation of side dishes, desserts, teas, and flavored liquors. Platycodi radix extract is also widely used as a dietary supplement for the treatment of pulmonary diseases and respiratory disorders. The saponins (platycosides) in Platycodi radix extract exhibit diverse pharmacological activities, including anti-obesity [1], anti-inflammatory [2, 3], anti-allergy [4], anti-oxidant [5], neuroprotective [6, 7], and antitumor effects [8]. Platycosides in Platycodi radix extract are composed of pentacyclic triterpenes with two side chains. One of these side chains comprises the β-glucose residue, which is linked by a glycosidic bond at C-3 in the aglycon, whereas the other side chain includes an oligosaccharide moiety (apiofuranosyl-xylopyranosyl-rhamnopyranosyl-arabinofuranosyl residue) attached to the ester linkage at C-28 (Fig. 1).

Deglycosylated saponins, which are obtained from the biotransformation of glycosylated saponins, exert stronger biological effects than their glycosylated forms [9, 10]. The commercial enzymes snailase [11], laminarinase [12], and cellulase [13] convert deapiosylated platycoside E (deapi-PE) and platycoside E (PE) into deapiosylated platycodin D (deapi-PD) and platycodin D (PD) via deapiosylated platycodin D_3_ (deapi-PD_3_) and platycodin D_3_ (PD_3_) by deglucosylation, respectively. The β-glucosidase from *Aspergillus usamii* converts PE into PD via PD_3_ [14]. A crude enzyme from *Aspergillus niger* is known to convert PD into deapiosylated dexylosylated platycodin D (deapi-dexyl-PD) [15], while the β-glucosidase from *Dictyoglomus turgidum* converts PD into deglucosylated PD (deglu-PD) [16]. However, there is no report on the biotransformation by the hydrolysis of the oligosaccharide moiety at the C-28 position of platycosides to date. Although 3-*O*-β-D-glucopyranosyl platycodigenin, 3-*O*-β-D-glucopyranosyl polygalacic acid, and 3-*O*-β-D-glucopyranosyl platyconic acid have been identified as the metabolites or saponins [17], no study has attempted in vitro enzymatic synthesis of 3-*O*-β-D-glucopyranosyl platycosides from glycosylated platycosides in Platycodi radix extract.

In the present study, glycosylated platycosides in Platycodi radix extract were biotransformed into deglycosylated platycosides by pectinase from *Aspergillus aculeatus*. After the biotransformation, the chemical structures of the deglycosylated platycosides were identified as 3-*O*-β-D-glucopyranosyl platycosides by LC/MS. In addition, the biotransformation pathways of glycosylated platycosides in Platycodi radix into 3-*O*-β-D-glucopyranosyl platycosides were investigated.

## Materials and Methods

### Materials

*P. grandiflorum* root was purchased from a local market (Republic of Korea). Platycoside standards, including deapi-PE, PE, deapi-PD, PD_3_, polygalacin D_3_, PD, and platyconic acid A, were purchased from Ambo Institute (Republic of Korea), while pectinase from *Aspergillus aculeatus* as the commercial enzyme Pectinex Ultra SP-L was obtained from Novozymes (Denmark).

### Preparation of Platycoside Standards

Platycodin A (PA), 3″-*O*-acetyl polygalacin D_3_, and deapi-PD_3_ standards were purified from glycosylated platycosides from Platycodi radix extract. To prepare 3-*O*-β-D-glucopyranosyl platycodigenin, 3-*O*-β-D-glucopyranosyl polygalacic acid, and 3-*O*-β-D-glucopyranosyl platyconic acid standards, the 3-*O*-β-D-glucopyranosyl platycoside product solutions were obtained from the reactions at 50°C with pectinase from *A. aculeatus* in 50 mM citrate-phosphate buffer (pH 5.0) containing 10 mg/ml enzyme and 1 mg/ml of reagent-grade PE, polygalacin D_3_, and platyconic acid A as substrates, respectively, after 24 h. Platycodi radix extract and 3-*O*-β-D-glucopyranosyl platycoside product solutions were separated with preparative high-performance liquid chromatography (Prep-HPLC) (Agilent, USA) equipped with a Hydrosphere C18 prep column (10 × 250 mm, 5 μm particle size; YMC, Japan), eluted with water at 30°C at a flow rate of 4.7 ml/min. The absorbance of the eluent was monitored at 203 nm, and collected using a fraction collector. The peak area ratios of PA, 3″-*O*-acetyl polygalacin D_3_, and deapi-PD_3_ obtained from the purification of glycosylated platycosides from Platycodi radix extract to total area in HPLC chromatograms were approximately 90%. The 3-*O*-β-D-glucopyranosyl platycosides showed 98% purity, as calculated from the ratio of the molar amount obtained after the purification of the products to the molar amount of the substrates.

### Extraction of Platycodi Radix

The dried root of *P. grandiflorum* (100 g) was suspended in 1 l of 99.8% (v/v) methanol and incubated at 80°C for 24 h. After incubation, the precipitates were eliminated with vacuum filtration through a filter with a pore size of 0.45 μm. The methanol was removed by evaporation, and the dried residue was dissolved in 1 l of water. The methanol-free solution was applied to a column containing Diaion HP-20 resin (500 mm × 12 mm). Other hydrophilic compounds and free sugars were removed by washing the column with water, and the adsorbed platycosides in the resin were extracted by sequentially eluting with 2 l of methanol at a flow rate of 0.5 ml/min. The methanol in the extracted platycosides was removed by evaporation, and the dried residue was dissolved in 1 l of water. The dissolved solution was diluted to 7.4% (w/v) Platycodi radix extract by adjusting the concentration of PE to 1.0 mg/ml, which was used for the biotransformation of platycosides.

### Biotransformation of Glycosylated Platycosides

The biotransformation into 3-*O*-β-D-glucopyranosyl platycodigenin, 3-*O*-β-D-glucopyranosyl polygalacic acid, and 3-*O*-β-D-glucopyranosyl platycconic acid in the presence of 10 mg/ml of pectinase from *A. aculeatus* were carried out at 50°C in 50 mM citrate-phosphate buffer (pH 5.0) with 1 mg/ml of reagent-grade PE, polygalacin D_3_, and platyconic acid A for 24 h, respectively. The biotransformation into 3-*O*-β-D-glucopyranosyl platycosides was performed at 50°C in 50 mM citrate-phosphate buffer (pH 5.0) containing 10 mg/ml enzyme and 7.4% (w/v) Platycodi radix extract containing 1 mg/ml PE, 0.05 mg/ml polygalacin D_3_, and 0.17 mg/ml platyconic acid A for 36 h.

### Analytical Methods

The reaction was terminated and exacted with *n*-butanol at a ratio of 1:1, resulting in the separation of components into *n*-butanol and water fractions. The *n*-butanol fraction was evaporated until complete dryness, and the dried residue was treated with methanol. The concentrations of platycosides were determined using the HPLC system (Agilent 1100) equipped with a Hydrosphere C18 column (4.6 × 150 mm, 5 μm particle size, YMC, Japan), which was eluted at 30°C with a gradient of acetonitrile from 10% to 40% (v/v) for 30 min, 40% to 90% for 30−45 min, 90% to 10% for 45−50 min, and 10% for 50−60 min at a flow rate of 1 ml/min. All platycosides were quantified from the calibration curves constructed using standard solutions of 0.2 to 0.8 mg/ml platycosides in triplicates.

LC/MS analysis of platycosides was performed to identify the chemical structures using a Thermo-Finnigan LCQ Deca XP Plus ion trap MS (Thermo Scientific, USA) at the NICEM (Seoul National University, Republic of Korea). The samples were ionized using electrospray ionization under the conditions of 275°C capillary temperature, 30 psi nebulizer gas, 5 kV ion source voltage, 46 V capillary voltage in positive mode, 15 V fragmentor voltage in negative ionization mode, 0.01 min average scan time, 0.02 min average time to change polarity, and 35% abundant precursor ions at collision energy.

## Results and Discussion

### HPLC Analysis for Biotransformation of Glycosylated Platycosides from Platycodi Radix Extract by Pectinase

**Fig. 2:**
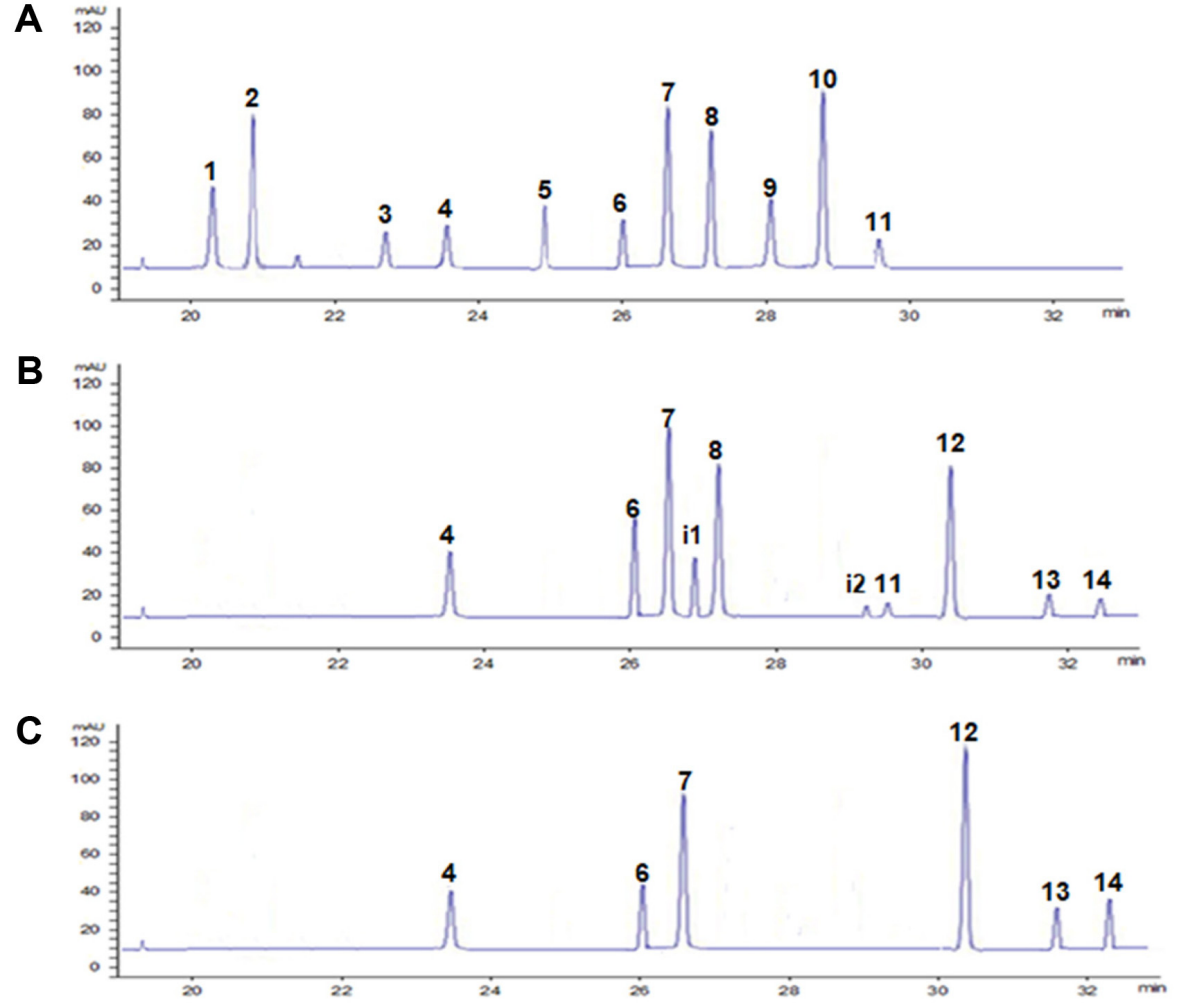
HPLC chromatograms from the biotransformation of platycosides in 7.4% (w/v) Platycodi radix extract with pectinase from *A. aculeatus*. (**A**) Platycosides from Platycodi radix extract. Deapi-platycoside E (**1**), platycoside E (**2**), deapi-platycodin D_3_ (**3**), platycodin D_3_ (**4**), polygalacin D_3_ (**5**), deapi-platycodin D (**6**), platycodin D (**7**), polygalacin D (**8**), 3″-*O*-acetyl polygalacin D_3_ (**9**), platycodin A (**10**), and platyconic acid A (**11**). (**B**) Platycosides at 12 h. Deapiplatycodin D_3_ (**3**), deapi-platycodin D (**6**), platycodin D (**7**), intermediate platycoside 1 (**i1**), polygalacin D (**8**), intermediate platycoside 2 (**i2**) polygalacin D (**9**), platyconic acid A (**11**), unknown product 1 (**12**), unknown product 2 (**13**), and unknown product 3 (**14**). (**C**) Platycosides at 36 h. Deapi-platycodin D_3_ (**3**), deapi-platycodin D (**6**), platycodin D (**7**), and unknown product 1 (**12**), unknown product 2 (**13**), and unknown product 3 (**14**). The biotransformation was performed at 50°C in 50 mM citrate-phosphate buffer (**pH 5.0**) containing 10 mg/ml enzyme and Platycodi radix extract containing 1 mg/ml PE, 0.05 mg/ml polygalacin D_3_, and 0.17 mg/ml platyconic acid A for 36 h.

 The glycosylated platycosides, namely, deapi-PE (**1**), PE (**2**), deapi-PD_3_ (**3**), PD_3_ (**4**), polygalacin D_3_ (**5**), deapi-PD (**6**), PD (**7**), polygalacin D (**8**), 3″-*O*-acetyl polygalacin D_3_ (**9**), PA (**10**), and platyconic acid A (**11**) from Platycodi radix extract were identified with HPLC at the same retention times of the standard platycosides (Fig. 2A). The biotransformation of the glycosylated platycosides from Platycodi radix extract into deglycosylated platycosides was performed with pectinase from *A. aculeatus*. In the HPLC chromatogram at 12 h, deapi-PD_3_ (**3**), deapi-PD (**6**), PD (**7**), polygalacin D (**8**), platyconic acid A (**11**), intermediate platycoside 1 (**i1**) intermediate platycoside 2 (**i2**), unknown product 1 (**12**), unknown product 2 (**13**), and unknown product 3 (**14**) were detected, whereas deapi-PE (**1**), PE (**2**), PD_3_ (**4**), polygalacin D_3_ (**5**), 3″-*O*-acetyl polygalacin D_3_ (**9**), PA (**10**) were disappeared (Fig. 2B). In the HPLC chromatogram at 36 h, polygalacin D (**8**), platyconic acid A (**11**), intermediate platycoside 1 (**i1**), and intermediate platycoside 2 (**i2**) were disappeared, whereas deapi-PD_3_ (**3**), deapi-PD (**6**), PD (**7**), unknown product 1 (**12**), unknown product 2 (**13**) and unknown product 3 (**14**) were detected (Fig. 2C). The reagent-grade PD_3_ (**4**) and deapi-PD (**6**) were completely converted into unknown product 1 (**12**). However, PD_3_ (**4**) and deapi-PD (**6**) in Platycodi radix extract were not much decreased in Fig. 2. The results may be due to the inhibition of the enzyme activity by other platycosides in Platycodi radix extract [18].

The total concentration of platycosides in 7.4% (w/v) Platycodi radix extract was 2.76 mg/ml, while the concentrations of PE, polygalacin D, and PD as the main compounds were 1.00, 0.80, and 0.27 mg/ml, respectively, corresponding to the contents of 36.2, 29.0, and 9.8% (w/w) to total platycosides, respectively (Table 1). After 36 h, the concentrations of unknown product 1 (**12**), unknown product 2 (**13**), and unknown product 3 (**14**) were 0.61, 0.21, 0.1 mg/ml, corresponding to the contents of 44.2, 15.2, and 7.2% (w/w), respectively.

### Identification of Unknown Products Obtained after Biotransformation of Glycosylated Platycosides from Platycodi Radix Extract by Pectinase

For identification of unknown products 1, 2, and 3, LC/MS analyses of these compounds were performed. The total molecular masses of unknown products 1, 2, and 3 were indicated by distinct peaks at mass per charge (*m/z*) 683.7, 667.2, and 697.6 [M+H]^+^, respectively, in the LC/MS spectra (Fig. 2). Based on LC/MS data, the unknown products 1, 2, and 3 were identified as 3-*O*-β-D-glucopyranosyl platycodigenin, 3-*O*-β-D-glucopyranosyl polygalacic acid, and 3-*O*-β-D-glucopyranosyl platyconic acid, respectively.

In the previous studies, the glycosylated platycoside PE was converted into PD by β-glucosidase of *A. niger* [14] and into deglu-PD by β-glucosidase of *D. turgidum* [16]. PD was converted into deapi-dexyl PD by crude enzyme of *A. niger* [15]. In this study, the biotransformation of glycosylated platycosides in Platycodi radix extract into 3-*O*-β-D-glucopyranosyl platycosides by hydrolyzing oligosaccharide moiety (apiofuranosyl-xylopyranosyl-rhamnopranosyl-arabinofuranosyl) at C-28 was first reported.

### Hydrolytic Pathways Involved in Biotransformation of Glycosylated Platycosides into 3-*O*-β-D-Glucopyranosyl Platycosides

To determine the pathways involved in the biotransformation of glycosylated platycosides in Platycodi radix extract by pectinase from *A. aculeatus*, we carried out the biotransformation of the reagent-grade glycosylated platycosides PE, polygalacin D_3_, and platyconic acid A. In the biotransformation, PE was completely converted into 3-*O*-β-D-glucopyranosyl platycodigenin via PD and deapi-PD (Figs. S1A-S1C), while polygalacin D_3_ was completely transformed into 3-*O*-β-D-glucopyranosyl polygalacic acid via polygalacin D and intermediate 1 (Figs. S2A-S2C). Platyconic acid A was completely converted into 3-*O*-β-D-glucopyranosyl platyconic acid via intermediate 2 (Figs. S3A-S3C).

Deapi-PD was confirmed as an intermediate formed during the biotransformation of PE into 3-*O*-β-D-glucopyranosyl platycodigenin with LC/MS. The total molecular mass of deapi-PD was indicated by the main peak at *m/z* 1093.3 [M+H]^+^ in the LC/MS spectrum. The fragment peaks resulted from the cleavage of xylose, rhamnose, and arabinose at C-28 and glucose at C-3, indicating that the intermediate was deapi-PD (Fig. S1D). The intermediates 1 and 2 were also identified by LC/MS. The total molecular mass of intermediates 1 and 2 were represented by peaks at *m/z* 1077.6 and 1107.3 [M+H]^+^, respectively. These results indicate that the intermediates 1 and 2 are deapi-polygalacin D and deapi-platyconic acid A (Figs. S2D and S3D). The fragment peaks of these compounds resulted from the cleavage of xylose, rhamnose, and arabinose at C-28 and glucose at C-3.

The biotransformation pathways of PE, polygalacin D_3_, and platyconic acid A in Platycodi radix extract were determined by HPLC analysis (Figs. S3-S5). The pathways involved in the biotransformation of other glycosylated platycosides in Platycodi radix extract, including deapi-PE, deapi-PD_3_, PD_3_, PA, and 3″-*O*-acetyl polygalacin D_3_, were investigated using reagent-grade platycosides in the HPLC chromatograms (Fig. S4). Deapi-PE, deapi-PD_3_, and PD_3_ as well as PA were completely converted into 3-*O*-β-D-glucopyranosyl platycodigenin via deapi-PD and deapi-PA, respectively. The compound 3″-*O*-acetyl polygalacin D_3_ was completely converted into 3-*O*-β-D-glucopyranosyl polygalacic acid via 3″-*O*-acetyl polygalacin D and deapi-3″-*O*-acetyl polygalacin D.

**Fig. 4:**
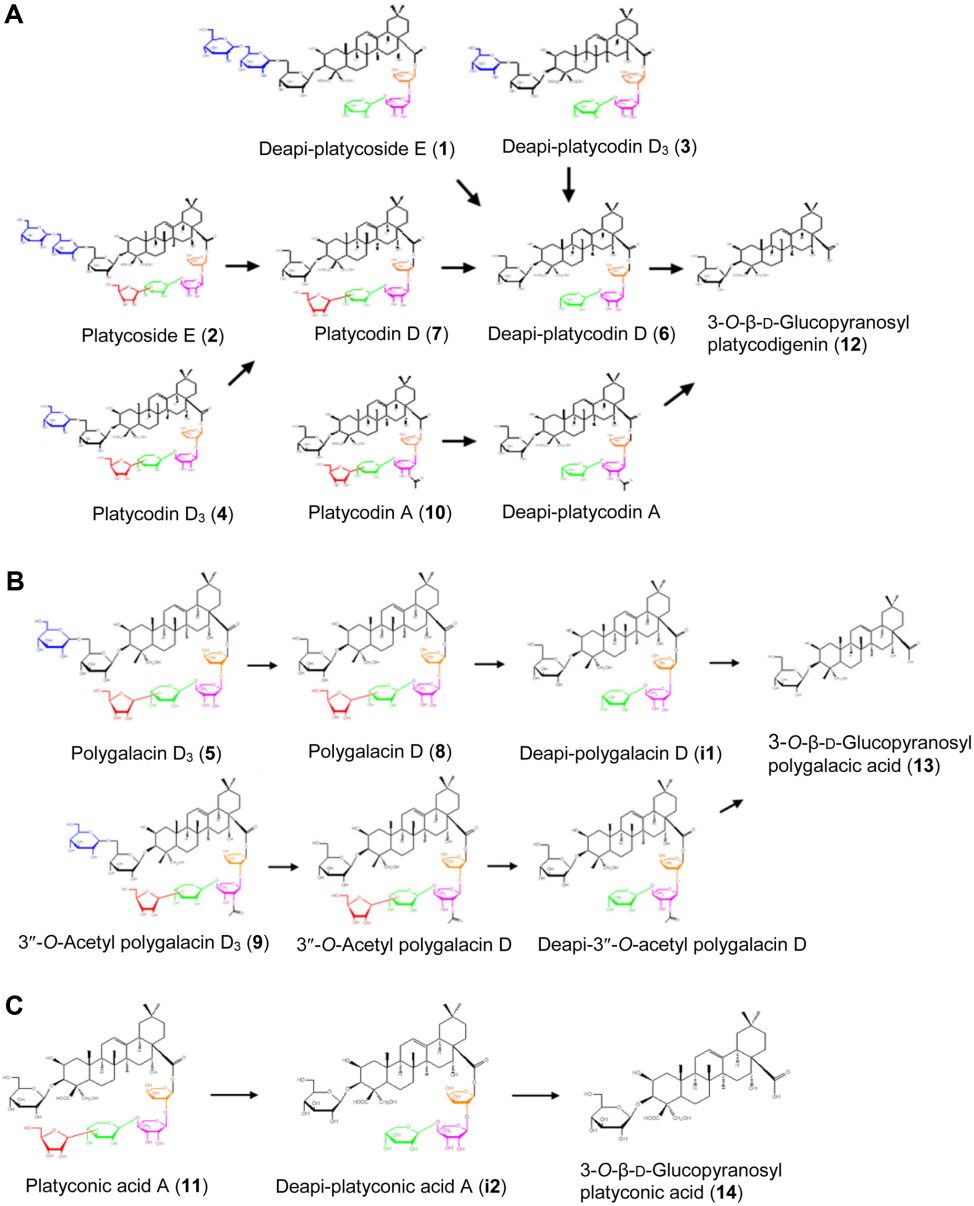
Pathways involved in the biotransformation of glycosylated platycosides from Platycodi radix extract into 3-*O*-β-D-glucopyranosyl platycosides with pectinase from *A. aculeatus*. (**A**) Pathways involved in the biotransformation of platycodigenin-type platycosides, including deapi-platycoside E, platycoside E, platycodin D_3_, and platycodin A, into 3-*O*-β-D-glucopyranosyl platycodigenin. (**B**) Pathways involved in the biotransformation of polygalacic acid-type platycosides, including polygalacin D_3_ and 3″-*O*-acetyl polygalacin D_3_, into 3-*O*-β-D-glucopyranosyl polygalacic acid. (**C**) Pathways involved in the biotransformation of platyconic acid-type platycoside, platyconic acid A, into 3-*O*-β-Dglucopyranosyl platyconic acid. Unnumbered platycosides were not found in the HPLC chromatograms of Fig. 2. However, they were confirmed with reagent-grade platycosides (Fig. S4).

Based on the HPLC data, the hydrolytic pathways of the three typical platycosides, including the platycodigenin-type platycosides deapi-PE (**1**), PE (**2**), deapi-PD_3_ (**3**), PD_3_ (**4**), deapi-PD (**6**), PD (**7**), and PA (**10**); polygalacic acid-type platycosides polygalacin D_3_ (**5**), polygalacin D (**8**), and 3″-*O*-acetyl polygalacin D_3_ (**9**); and the platyconic acid-type platycoside platyconic acid A (**11**) of major glycosylated platycosides in Platycodi radix extract into 3-*O*-β-D-glucopyranosyl platycodigenin (**12**), 3-*O*-β-D-glucopyranosyl polygalacic acid (**13**), and 3-*O*-β-D-glucopyranosyl platyconic acid (**14**), respectively, by pectinase from *A. aculeatus* were newly established as shown in Fig. 4. In the hydrolytic pathways, the enzyme hydrolyzed the glucose molecules, leaving one glucose residue at C-3 and the oligosaccharide moiety (apiofuranosyl-xylopyranosyl-rhamnopyranosyl-arabinofuranosyl residue) at C-28.

The biotransformation pathway of PE into PD by β-glucosidase of *A. niger* [14] and that of PE into deglu-PD by β-glucosidase of *Caldicellulosiruptor bescii* [19] were previously reported. In addition, the biotransformation pathways of glycosylated platycosides by human intestinal bacteria were suggested [20]. However, the biotransformation pathways of glycosylated platycosides in Platycodi radix extract into 3-*O*-β-D-glucopyranosyl platycosides by pectinase was first identified in this study.

### Biotransformation of Platycoside E, Polygalacin D_3_, Platyconic Acid A, and Glycosylated Platycosides in Platycodi Radix Extract into 3-*O*-β-D-Glucopyranosyl Platycosides

**Fig. 5:**
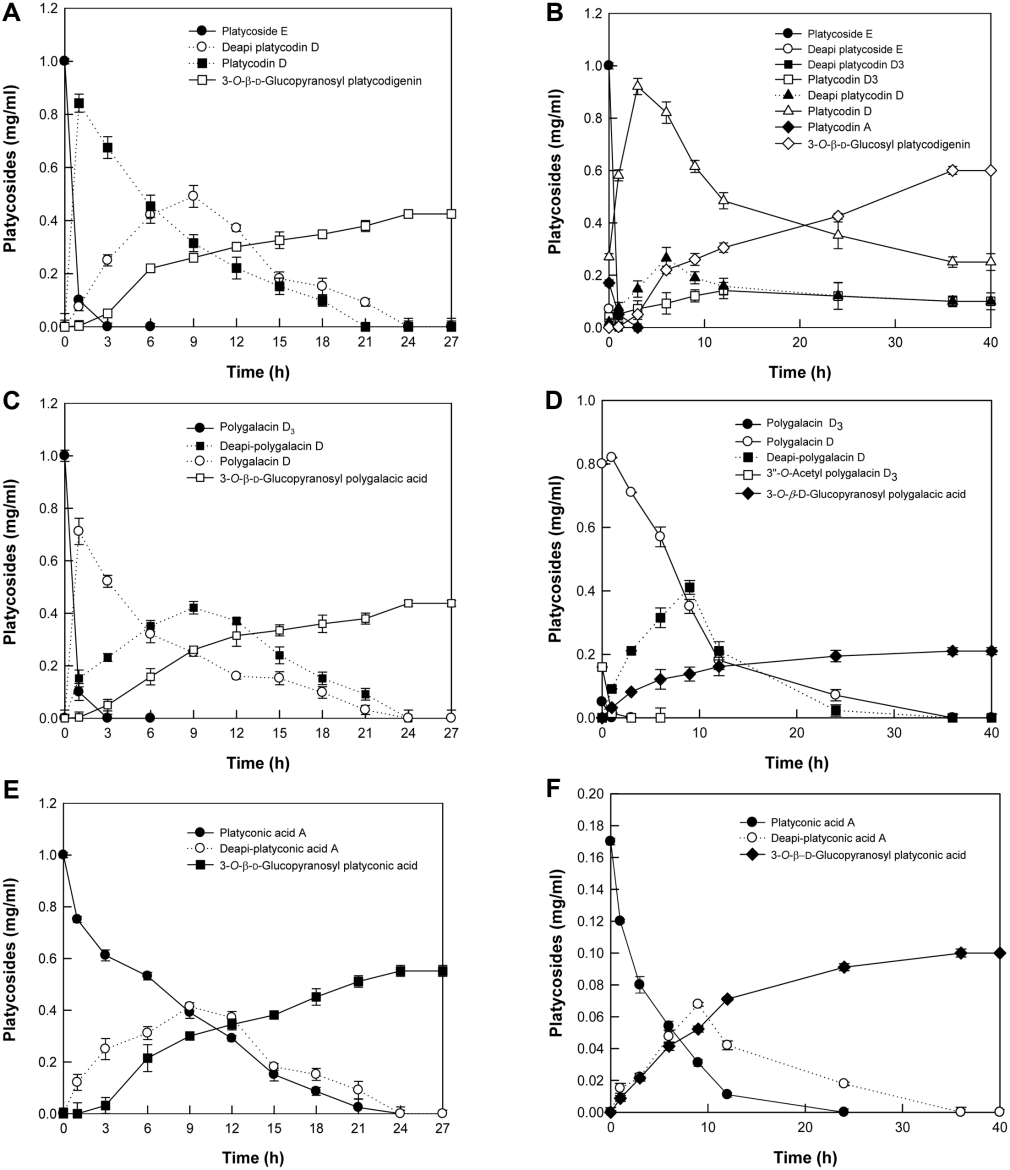
Biotransformation of reagent-grade platycosides and glycosylated platycosides from Platycodi radix extract into 3-*O*-β-D-glucopyranosyl platycosides by pectinase from *A. aculeatus*. (**A**) Biotransformation of reagent-grade PE. (**B**) Biotransformation of platycodigenin-type platycosides from Platycodi radix extract. (**C**) Biotransformation of reagent-grade polygalacin D_3_. (**D**) Biotransformation of polygalacic acid-type platycosides from Platycodi radix extract. (**E**) Biotransformation of reagent-grade platyconic acid A. (**F**) Biotransformation of platyconic acid-type platycosides from Platycodi radix extract.

The time-course reactions for the biotransformation of reagent-grade PE as a platycodigenin-type platycoside were performed by pectinase from *A. aculeatus*. After 24 h, the enzyme completely converted 1 mg/ml PE into 0.42 mg/ml 3-*O*-β-D-glucopyranosyl platycodigenin via PD and deapi-PD (Fig. 5A). The quantitative biotransformation of platycodigenin-type platycosides in Platycodi radix extract into 3-*O*-β-D-glucopyranosyl platycodigenin was also performed. The enzyme converted platycodigenin-type platycosides, including 0.07 mg/ml deapi-PE, 1.0 mg/ml PE, 0.01 mg/ml deapi-PD_3_, 0.04 mg/ml PD_3_, 0.02 mg/ml deapi-PD, 0.27 mg/ml PD, and 0.17 mg/ml PA, in Platycodi radix extract into 0.61 mg/ml 3-*O*-β-D-glucopyranosyl platycodigenin as the main product and 0.1 mg/ml PD_3_, 0.11 mg/ml deapi-PD, and 0.25 mg/ml PD as intermediates after 36 h (Fig. 5B).

The quantitative production of 3-*O*-β-D-glucopyranosyl polygalacic acid was attempted using the reagent-grade polygalacic acid-type polygalacin D_3_. After 24 h, the enzyme completely converted 1 mg/ml polygalacin D_3_ into 0.43 mg/ml 3-*O*-β-D-glucopyranosyl polygalacic acid via polygalacin D and deapi-polygalacin D (Fig. 5C). The enzyme was also used for the quantitative biotransformation of polygalacic acid-type platycosides in Platycodi radix extract, including 0.05 mg/ml polygalacin D_3_, 0.8 mg/ml polygalacin D, and 0.16 mg /ml 3″-*O*-acetyl polygalacin D_3_, which were converted into 0.21 mg/ml 3-*O*-β-D-glucopyranosyl polygalacic acid as a single product via 3″-*O*-acetyl polygalacin D and deapi-polygalacin D after 36 h (Fig. 5D).

The time-course reactions for the biotransformation of reagent-grade platyconic acid A as a platyconic acid-type platycoside were performed. The enzyme completely converted 1 mg/ml platyconic acid A into 0.55 mg/ml 3-*O*-β-D-glucopyranosyl platyconic acid after 24 h via deapi-platyconic acid A (Fig. 5E). The time-course reactions for the biotransformation of platyconic acid A in Platycodi radix extract into 3-*O*-β-D-glucopyranosyl platyconic acid were also performed. The enzyme completely converted 0.17 mg/ml platyconic acid A as a platyconic acid-type platycoside in the Platycodi radix extract into 0.1 mg/ml 3-*O*-β-D-glucopyranosyl platyconic acid as a single product via deapi-platyconic acid A after 36 h (Fig. 5F).

The quantitative enzymatic production of 3-*O*-β-D-glucopyranosyl platycosides was carried out for the first time in the present study. Although the biotransformation of reagent-grade platycosides resulted in the complete conversion of the substrate into 3-*O*-β-D-glucopyranosyl platycosides after 24 h, we failed to observe the complete conversion of the platycosides in Platycodi radix extract after 24 h. The retardation effect may be attributed to the inhibition of the enzyme activity by other platycosides in the extract [18].

Saponins have been reported to improve in functionality as they are deglycosylated . For example, the anti-inflammatory activities of the platycodigenin-type platycosides followed the order deglu PD (three glycosides) > PD (four glycosides) > PD_3_ (six glycosides) > PE (seven glycosides) [16] and as an antioxidant activity, the peroxynitrite-scavenging capacities followed the order platycodigenin (no glycosides) > deapi-PE > PD > PE [21]. Thus, 3-*O*-β-D-glucopyranosyl platycosides (one glycoside) are expected to have high functionality and further study is needed in the functional properties of the deglycosylated platycosides.

In summary, the pectinase from *A. auleatus* converted glycosylated platycosides into the deglycosylated 3-*O*-β-D-glucopyranosyl platycosides by the hydrolysis of the glucose molecules at C-3, leaving one glucose residue, and the hydrolysis of the oligosaccharide moiety (apiofuranosyl-xylopyranosyl-rhamnopyranosyl-arabinofuranosyl residue) at C-28. This is the first report on the quantitative enzymatic production of 3-*O*-β-D-glucopyranosyl platycosides and the establishment of the biotransformation pathways.

## Supplemental Materials

Supplementary data for this paper are available on-line only at http://jmb.or.kr.

## Figures and Tables

**Fig. 3:**
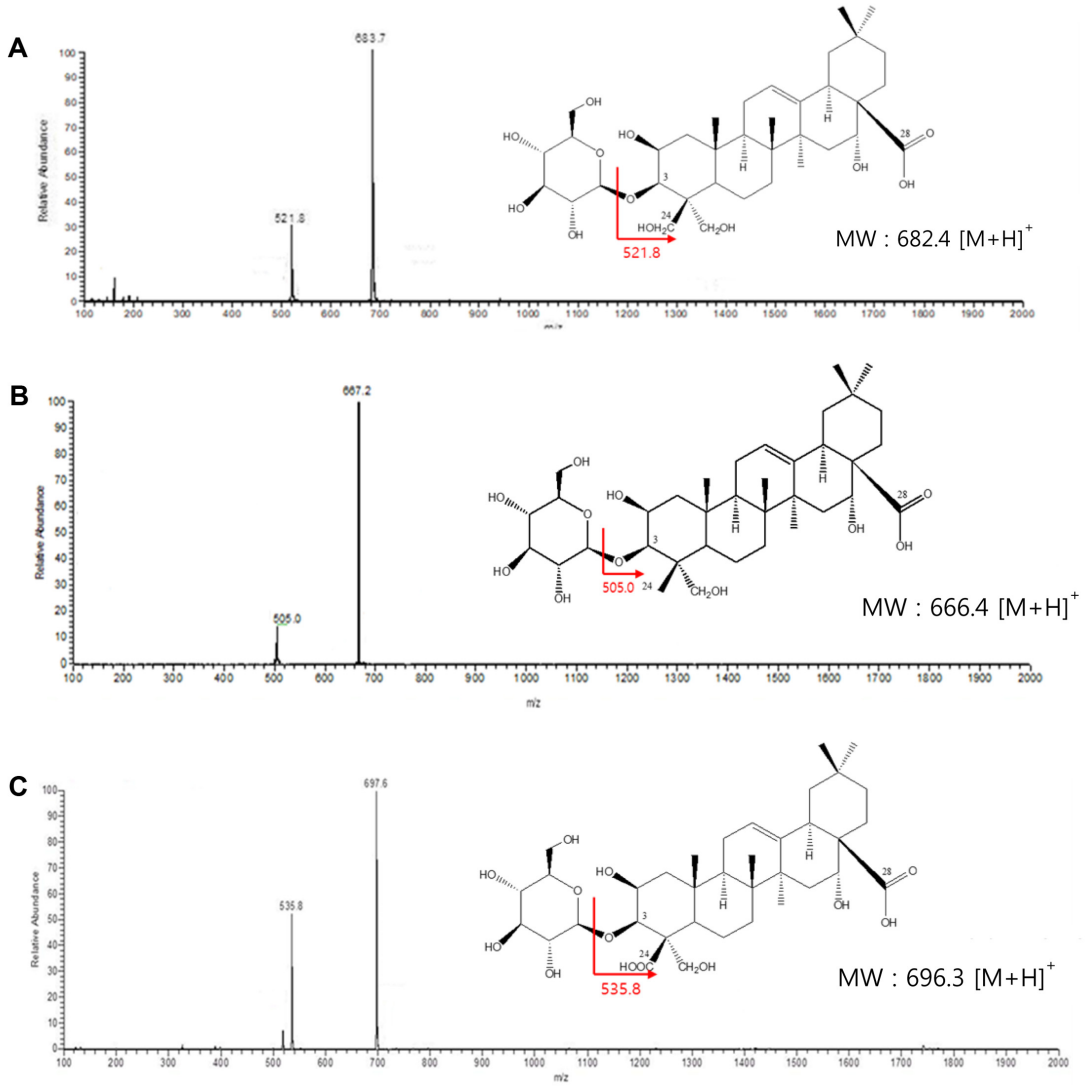
LC/MS chromatograms of 3-*O*-β-D-glucopyranosyl platycosides. (**A**) 3-*O*-β-D-Glucopyranosyl platycodigenin. (**B**) 3-*O*-β-D-Glucopyranosylpolygalacic acid. (**C**) 3-*O*-β-D-Glucopyranosylplatyconic acid.

**Table 1:** Platycoside content in 7.4% (w/v) Platycodi radix extract before and after biotransformation by pectinase.

No.	Platycoside	Before reaction		After reaction	
	
		Content (%, w/w)	Concentration (mg/ml)	Content (%, w/w)	Concentration (mg/ml)
1	Deapi-platycoside E	2.53	0.07	0	0
2	Platycoside E	36.23	1.00	0	0
3	Ddeapi-platycodin D_3_	0.36	0.01	0	0
4	Platycodin D_3_	1.45	0.04	7.24	0.10
5	Polygalacin D_3_	1.81	0.05	0	0
6	Deapi-platycodin D	0.72	0.02	7.97	0.11
7	Platycodin D	9.78	0.27	18.1	0.25
8	Polygalacin D	28.98	0.80	0	0
9	3″-*O*-Acetyl polygalacin D_3_	5.80	0.16	0	0
10	Platycodin A	6.15	0.17	0	0
11	Platyconic acid A	6.15	0.17	0	0
i1	Iintermediate 1	ND	ND	ND	ND
i2	Intermediate 2	ND	ND	ND	ND
12	Unknown product 1	ND	ND	44.20	0.61
13	Unknown product 2	ND	ND	15.22	0.21
14	Unknown product 3	ND	ND	7.24	0.10
	Total	100	2.76	100	1.38

ND: not detected.
