# Supplementary material for: Biotransformation of Glycosylated Saponins in Balloon Flower Root Extract into 3-O-β-D-Glucopyranosyl Platycosides by Deglycosylation of Pectinase from Aspergillus aculeatus
Source: J Microbiol Biotechnol. 2020 Mar 13;30(6):946–54. doi: 10.4014/jmb.2001.01041 (PMC9728355; doi:10.4014/jmb.2001.01041)
Supplement: Supplementary file 1 [file JMB-30-6-946-supple.pdf]

J. Microbiol. Biotechnol.
https://doi.org/10.4014/jmb.2001.01041

# Supporting information

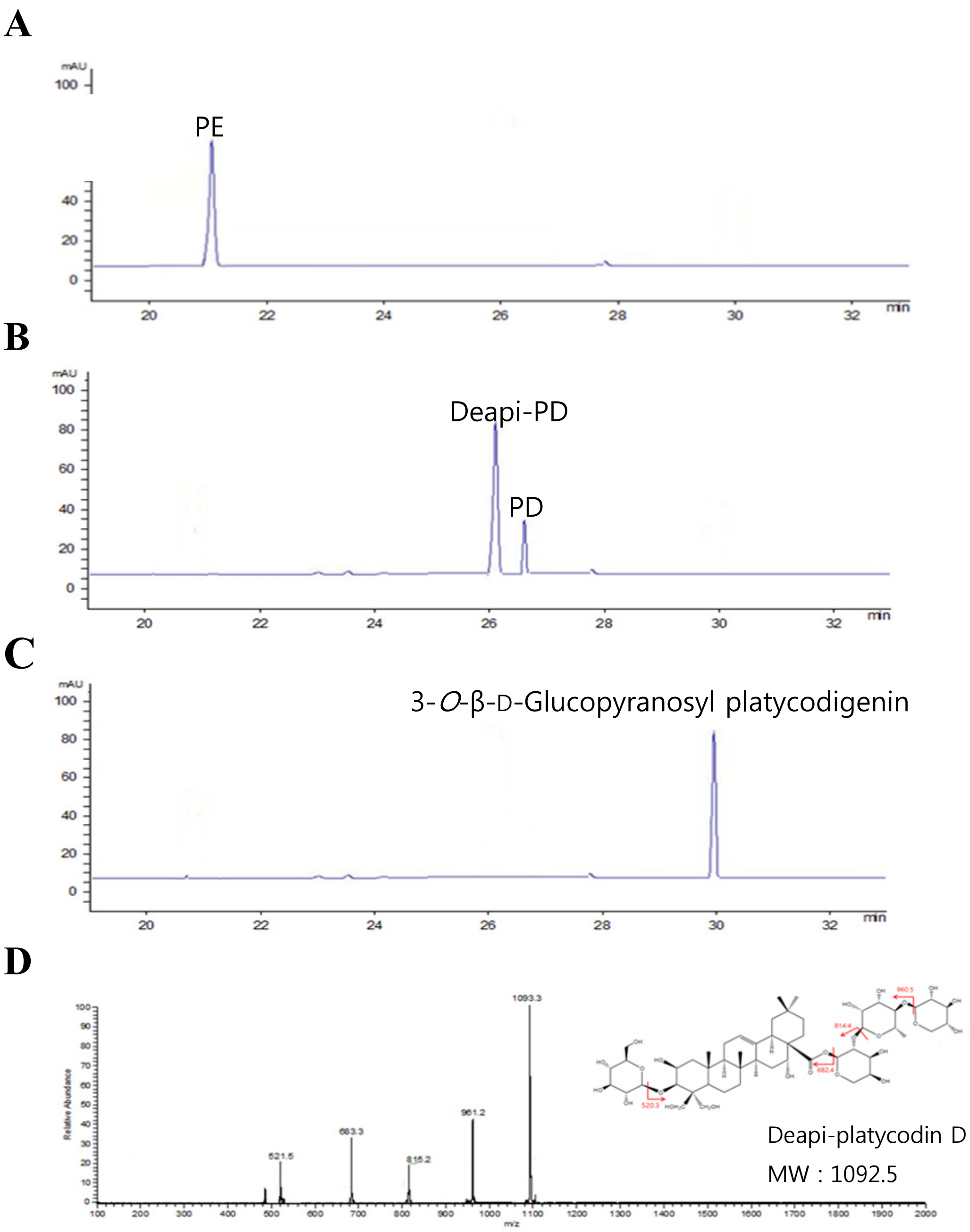


**Fig. S1.** HPLC chromatograms during the biotransformation of platycoside E in Platycodi radix extract by pectinase from *A. aculeatus*. The reactions were performed in 50 mM citrate-phosphate buffer pH 5.0 containing 10 mg/ml enzyme and 1 mg/ml PE at 50°C (A) PE in Platycodi radix extract. (B) Deapi-PD and PD at 12 h of reaction time during the biotransformation. (C) 3-*O*-β-D-glucopyranosyl platycodigenin at 24 h of reaction time during the biotransformation. (D) LC/MS data of the intermediate deapi-PD.

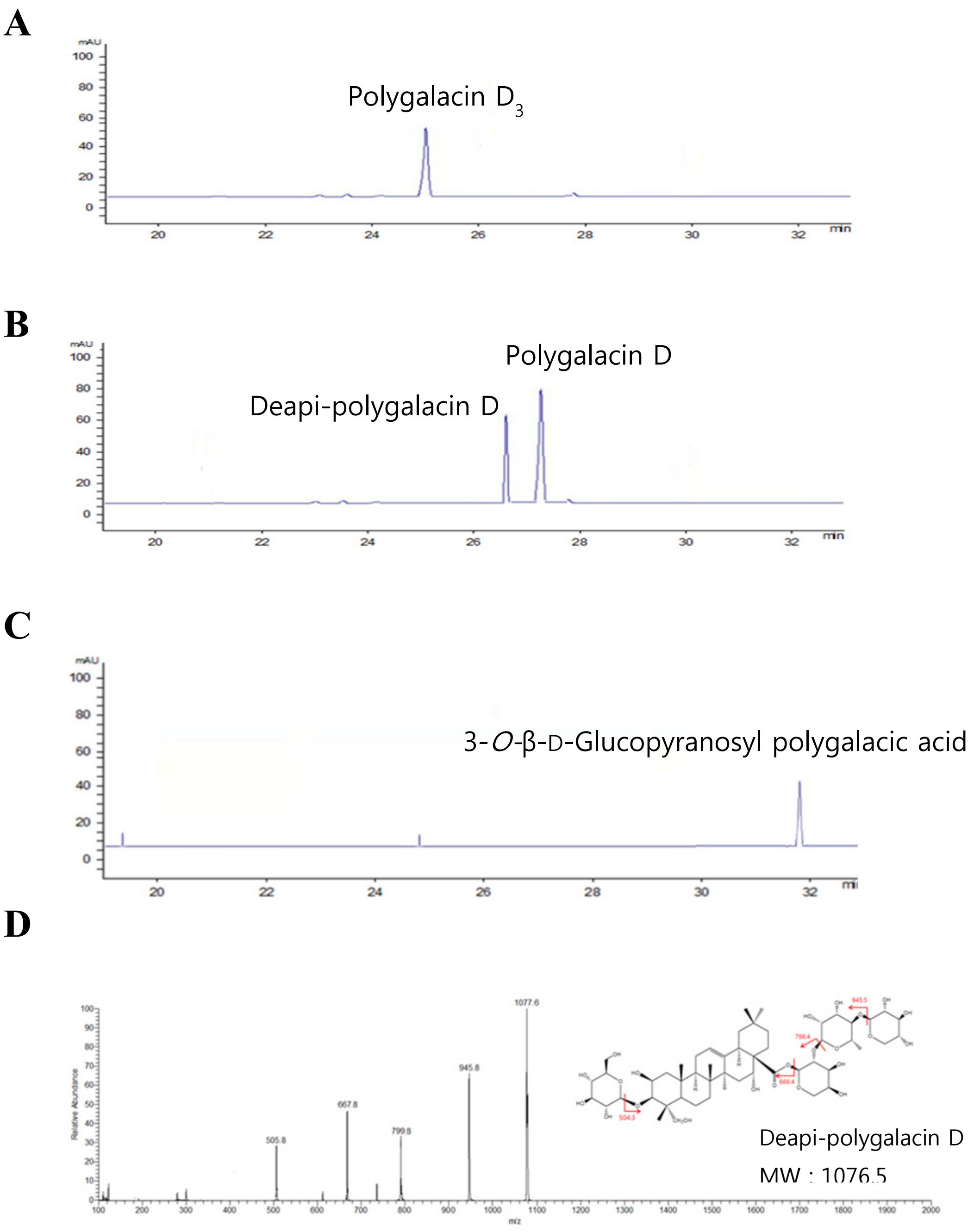


**Fig. S2.** HPLC chromatograms during the biotransformation of polygalacin $D_3$ in Platycodi radix extract by pectinase from *A. aculeatus*. The reactions were performed in 50 mM citrate-phosphate buffer pH 5.0 containing 10 mg/ml enzyme and 1 mg/ml polygalacin $D_3$ at 50°C. (A) Polygalacin $D_3$ in Platycodi radix extract. (B) Deapi-polygalacin D and polygalacin D were produced at 12 h of reaction time during the biotransformation. (C) 3-*O*-β-D-glucopyranosyl polygalacic acid at 24 h of reaction time during the biotransformation (D) LC/MS data of the intermediate deapi-polygalacin D.

A

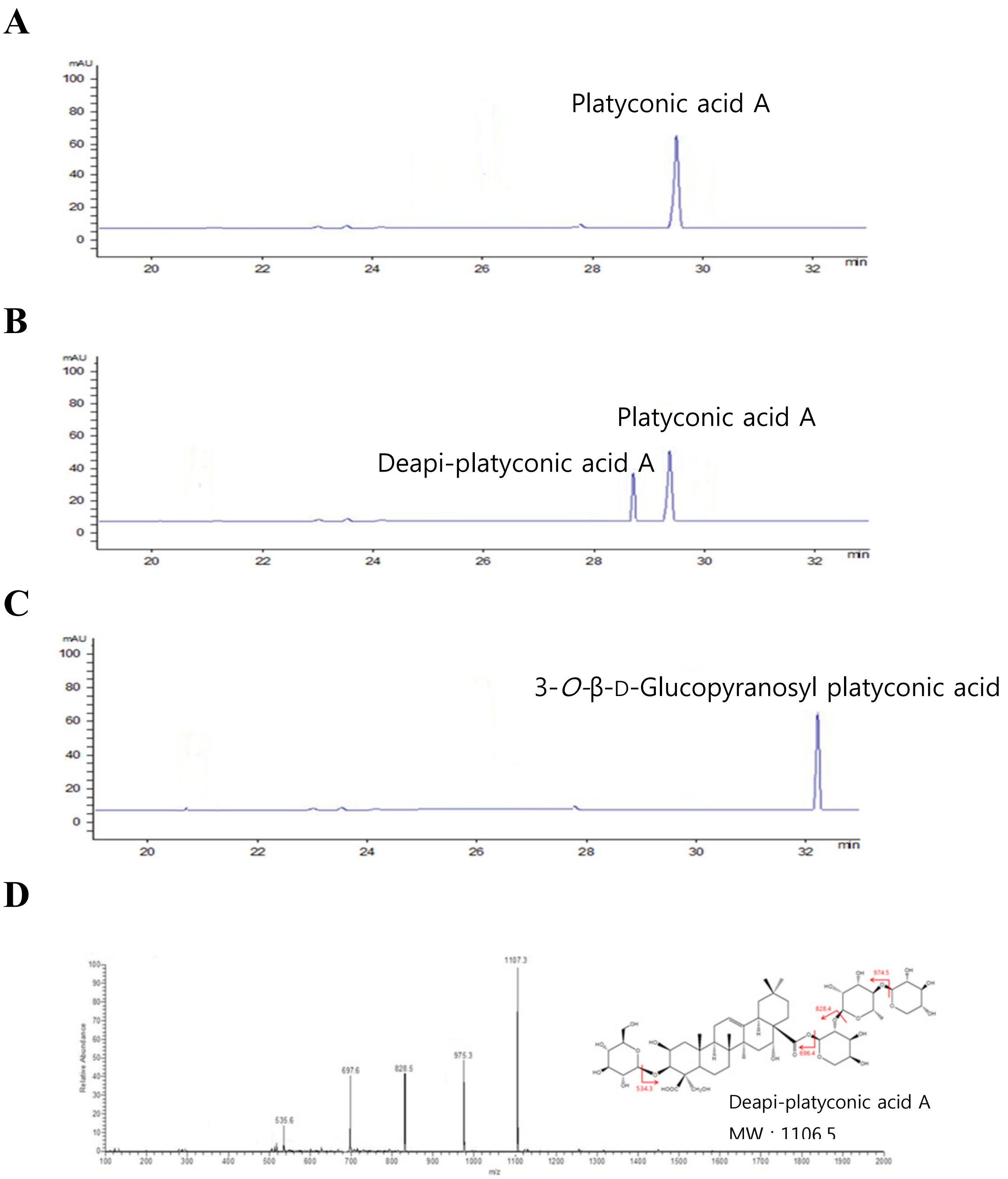


**Fig. S3**. HPLC chromatograms during the biotransformation of platyconic acid A in Platycodi radix extract by pectinase from *A. aculeatus*. The reactions were performed in 50 mM citrate-phosphate buffer pH 5.0 containing 10 mg/ml enzyme and 1 mg/ml platyconic acid A at 50°C. (A) Platyconic acid A in Platycodi radix extract. (B) Deapi platyconic acid A at 12 h of reaction time during the biotransformation. (C) 3-*O*-β-D-glucopyranosyl platyconic acid at 24 h of reaction time during the biotransformation. (D) LC/MS data of the intermediate deapi-platyconic acid A.

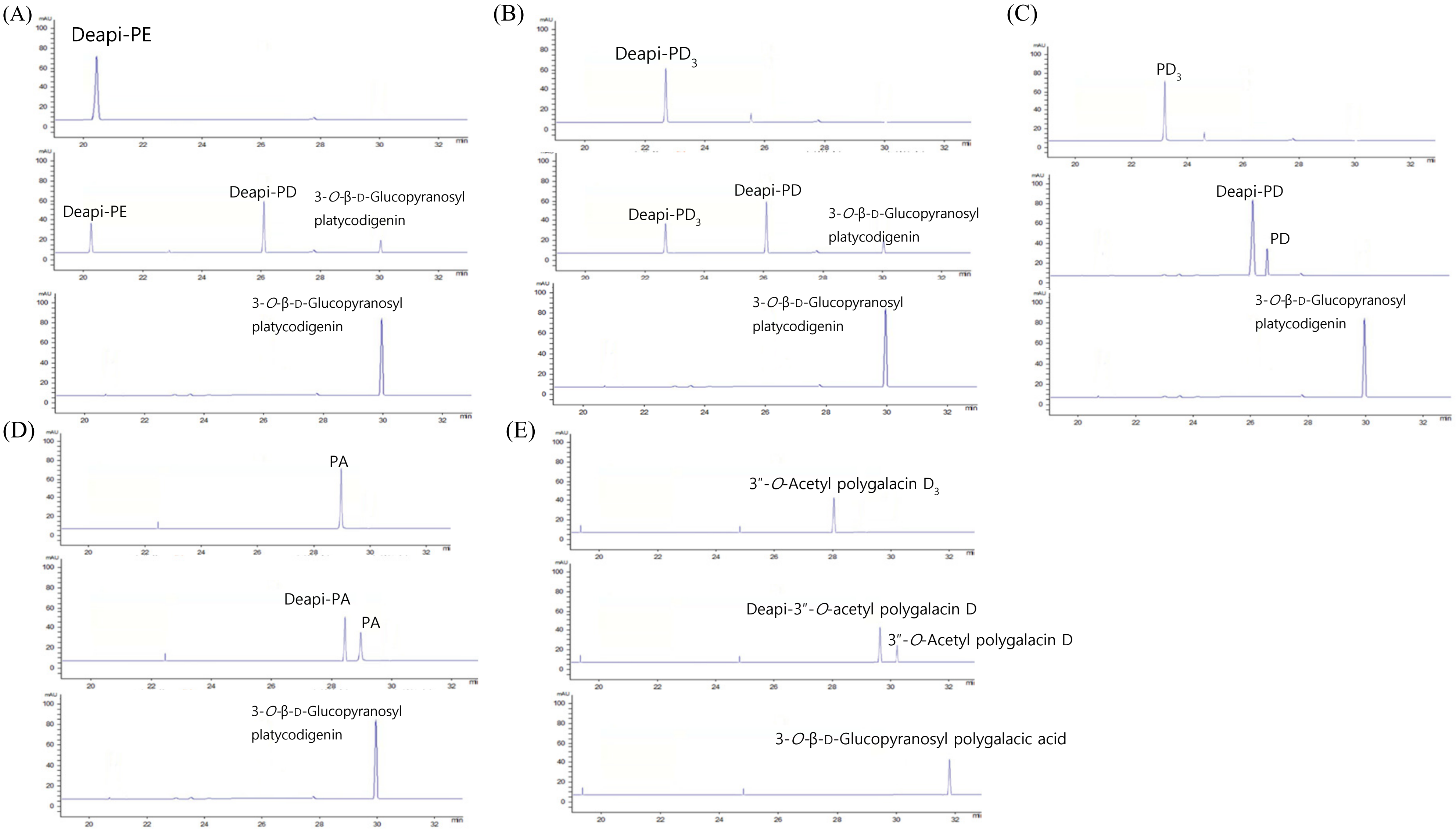


**Fig. S4**. HPLC chromatogram of platycosides in Platycodi Radix extract. The reactions were performed in 50 mM citrate-phosphate buffer pH 5.0 containing 10 mg/m enzyme and 1 mg/ml each of platycosides at 50°C. (A) HPLC chromatogram changes of deapi-PE during (A) 0 h, 12 h, and 24 h reaction. (B) HPLC chromatogram changes of deapi-$PD_3$ during (A) 0 h, 12 h, and 24 h reactions. (C) HPLC chromatogram changes of $PD_3$ during (A) 0 h, 12 h, and 24 h reaction. (D) HPLC chromatogram changes of PA during (A) 0 h, 12 h, and 24 h reactions. (E) HPLC chromatogram changes of 3″-*O*-acetyl polygalacin $D_3$ during (A) 0 h, 12 h, and 24 h reactions.
